# Central and peripheral contributions of T-type calcium channels in pain

**DOI:** 10.1186/s13041-022-00923-w

**Published:** 2022-05-02

**Authors:** Erika K. Harding, Gerald W. Zamponi

**Affiliations:** Department of Physiology and Pharmacology, Hotchkiss Brain Institute, Alberta Children’s Hospital Research Institute, University of Calgary, Calgary, AB T2N 4N1 Canada

**Keywords:** T-type, Pain, CACNA1H, Ca_v_3.2, Ubiquitination, Analgesia, Glycosylation

## Abstract

Chronic pain is a severely debilitating condition that reflects a long-term sensitization of signal transduction in the afferent pain pathway. Among the key players in this pathway are T-type calcium channels, in particular the Ca_v_3.2 isoform. Because of their biophysical characteristics, these channels are ideally suited towards regulating neuronal excitability. Recent evidence suggests that T-type channels contribute to excitability of neurons all along the ascending and descending pain pathways, within primary afferent neurons, spinal dorsal horn neurons, and within pain-processing neurons in the midbrain and cortex. Here we review the contribution of T-type channels to neuronal excitability and function in each of these neuronal populations and how they are dysregulated in chronic pain conditions. Finally, we discuss their molecular pharmacology and the potential role of these channels as therapeutic targets for chronic pain.

## Introduction

Calcium concentration within neurons is tightly regulated, with resting intracellular calcium typically maintained in the nanomolar range [1]. Voltage-gated calcium channels (VGCCs) allow for brief, but substantial increases in calcium concentration upon membrane depolarization. These channels are present on both presynaptic and postsynaptic neuronal membranes, where they can contribute to discrete compartmental calcium events and thus shape the communication and excitability of neurons [2–4]. VGCCs allow for both rapid calcium-dependent processes such as presynaptic vesicle release and dendritic calcium spikes, as well as activation of downstream signaling pathways. This includes calcium-dependent regulation of gene expression as observed with long-term plasticity of brain and spinal cord synapses, and neuronal growth and proliferation [5–8].

**Fig. 1:**
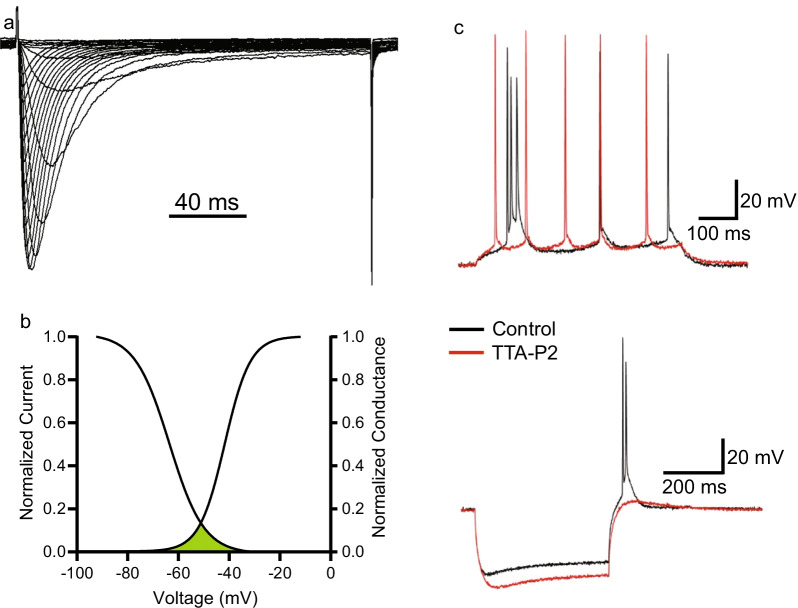
Electrophysiological properties of T-type channels. **a** Typical inward current recorded from T-type channels during an IV-curve. Note that inward current in response to depolarization is rapidly activating and rapidly inactivating. **b** Typical steady state activation and inactivation curves for T-type channels. Current activation occurs near − 60 mV and reaches peak around − 20 mV. Overlap of activation and inactivation curves reveals a significant window current between -60 and -40 mV. **c** Adapted with permission from Joksimovic et al. [33]. Perfusion of 10 µM TTA-P2 significantly reduces burst firing in response to current injection (top) and rebound bursting (bottom) in subicular neurons, revealing the contribution of T-type channels to activity in these neurons

The pore-forming α1 subunit of VGCCs can be encoded by ten different genes, producing ten distinct channels. These channels can then be separated functionally by their differing activation voltages, kinetics, and pharmacology [9]. The T-type calcium channel family possesses the lowest amino acid homology compared to the other families, and perhaps not surprisingly, this family has the highest divergence in physiological characteristics as well. T-type channels exhibit rapidly inactivating or transient current and activate at the most hyperpolarized voltage of any other VGCC in neurons at approximately − 60 mV, and they are thus termed low voltage activated (LVA) VGCCs (Fig. 1a, b) [10–12]. Conversely, all other VGCCs activate at − 40 mV or higher, and are therefore categorized as high voltage activated (HVA).

The T-type calcium channel α1 subunits are represented by three different genes (CACNA1G, CACNA1H, and CACNA1I) encoding Ca_v_3.1, Ca_v_3.2, and Ca_v_3.3 channels, respectively. These three T-type channels are highly similar, but possess slightly differing activation voltages, as well as differing sensitivity to inhibition by large, divalent cations (namely nickel, cadmium, and zinc) and certain T-type channel antagonists [7, 13, 14]. Whereas HVA channels require the presence of other, obligatory subunits for correct function (α2δ, β, and γ1), T-type channels function effectively as a single α1 subunit [15, 16].

The unique, hyperpolarized activation voltage of T-type channels means that they can be activated by subthreshold stimuli including synaptic potentials [17, 18]. In addition to this, a portion of T-type channels are tonically inactivated at resting membrane potential, and thus cannot be recruited during depolarization. Recovering these channels from inactivation via a brief hyperpolarization prior to depolarization results in a greatly increased inward T-type current, often sufficient to recruit voltage-gated sodium channels (VGSCs), thereby initiating action potential firing in what is referred to as a rebound burst [19–21]. It is this interplay between the activation and inactivation voltages of T-type channels that creates a window current around the resting membrane potential, such that a fraction of channels can be transiently active at rest (Fig. 1b) [22, 23]. In addition to the capacity to initiate rebound burst firing, T-type channels also contribute to the excitability of neurons through low-threshold dendritic calcium spikes [8, 24, 25] and action potential afterdepolarizations [26–28]. Inhibition or genetic knock-out or knockdown of T-type channels in many types of neurons is known to reduce neuronal excitability (Fig. 1c) [24, 26, 29–34].

While T-type channels are not present in every neuron, they are abundantly present within thalamocortical neurons, where they are known to contribute to the development of seizure disorders [35, 36] and within cortical and hippocampal pyramidal neurons where they may contribute to synaptic plasticity [37–40]. Similarly, within nociceptive circuitry including primary afferent neurons, the superficial dorsal horn, and within the brain T-type channel expression and regulation of excitability is thought to play a role in the development and maintenance of chronic pain [41–44].

Perturbations of nociceptive circuitry are common characteristics of chronic pain, which affects approximately 20–25% of all adults in North America. Chronic pain is a broad term encompassing any patient with pain that lasts for over three months and is roughly divided into inflammatory and neuropathic pain types, with each being caused by a variety of diseases, disorders, or even as a side effect of medications such as those used during chemotherapy. An abundance of evidence now indicates that hyperexcitability of both primary afferent neurons and spinal cord neurons is a major driver of chronic pain symptoms [42, 45–47]. Emerging evidence indicates that the brain circuitry involved in the processing of pain is also altered in chronic pain conditions with a shift towards hyperexcitability and a loss of descending inhibition of pain [44, 48–53]. Thus, a common goal for treatment of chronic pain is finding a way to dampen the hyperexcitability of these pain processing circuits without resulting in deleterious side effects. Here we review the evidence for a role of T-type channels in peripheral and central nociceptive circuitry, including within the spinal cord and brain, and highlight their potential utility as therapeutic targets for treatment of pain.

## Evidence for the presence of T-type channels in primary afferent neurons and their modulation in chronic pain conditions

Primary afferent neurons innervate our skin, tissues, and organs, providing the central nervous system with critical somatosensory information. Primary afferent neurons that carry nociceptive information are typically either unmyelinated (C fibers) or very lightly myelinated (Aδ fibers), and it is at these free nerve endings that nociceptive stimuli are first transduced into electrical output in the form of action potential firing [47]. This action potential then travels along the primary afferent axon into its soma which resides within the dorsal root ganglia (DRG), and into the dorsal horn.

T-type channels were first noted within primary afferent neurons as a low threshold activated calcium current, and some of the first characterizations of T-type channels were performed in cultured primary afferent neurons [10, 54]. Use of immunofluorescence, western blots, and genetic tools has confirmed that in rodents the Ca_v_3.2 channel is the predominant subtype present in somata of primary afferent neurons [55, 56] with significant expression of Ca_v_3.1 and Ca_v_3.3 as well [55]. T-type channels localized to primary afferent somata contribute to neuronal excitability, as loss or block of these channels reduces action potential firing during electrophysiological recordings [26, 34, 57]. Conversely, increasing T-type channel expression decreases threshold for action potential firing and promotes burst firing [21, 58]. This represents a potential mechanism through which upregulation of T-type channels in the DRG may contribute to chronic pain.

**Fig. 2:**
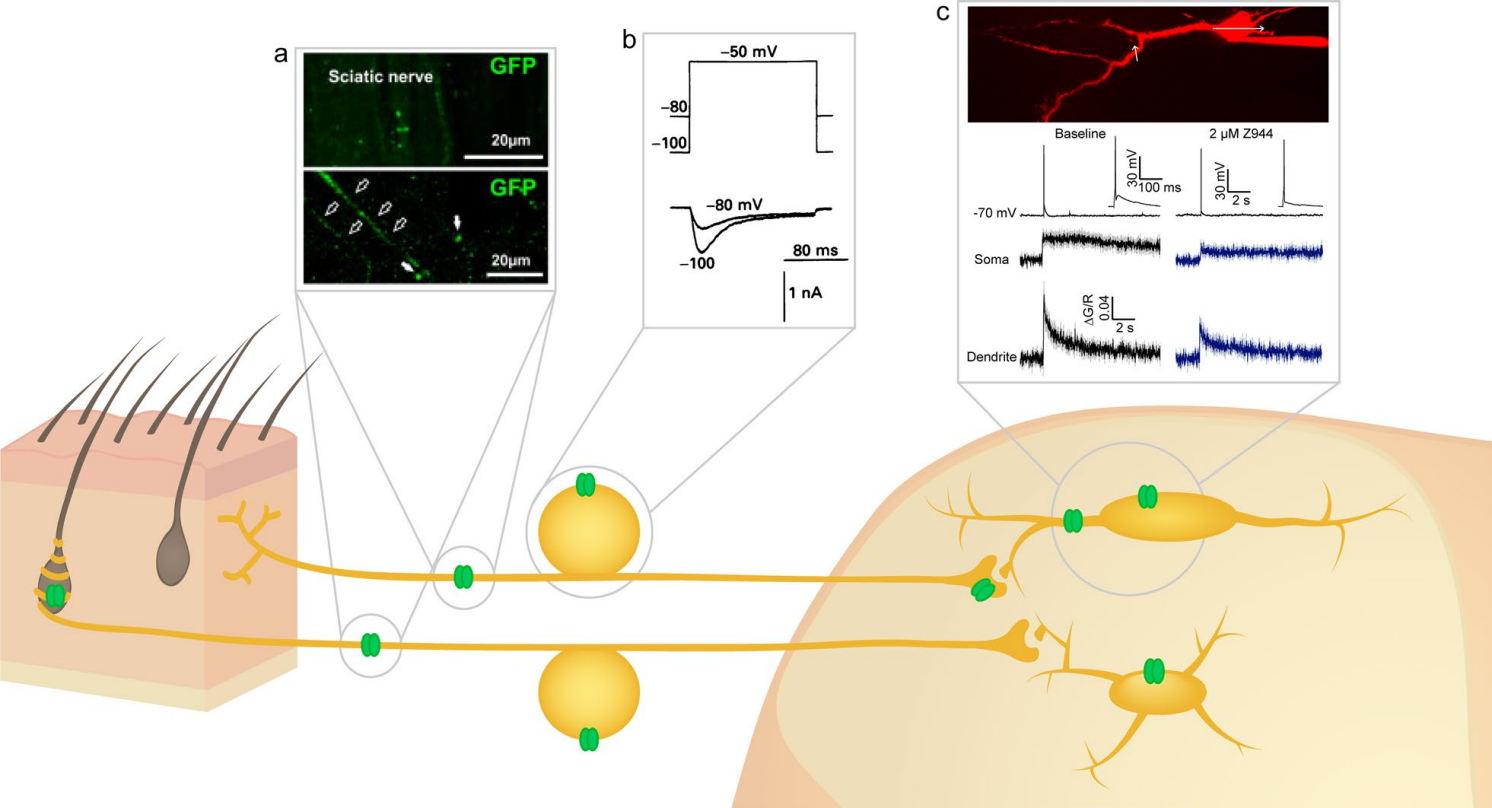
Locations of T-type channels in the ascending afferent pain pathway. In rodents, T-type channels (green) are present surrounding D-hair mechanoreceptors, on both C and Aδ afferent fibers, on C and Aδ DRG somata, on C fiber presynaptic terminals, and on both lamina I and II neurons within the superficial dorsal horn. Insets indicate key experiments defining the presence of T-type channels in different compartments. **a** Expression of Cav3.2 fused to GFP in C and Aδ mouse sciatic nerve fibers. Adapted with permission from Francois et al. [67]. **b** Presence of low voltage-activated current in small diameter rat DRG neurons. Adapted with permission from Scroggs and Fox [59]. **c** T-type calcium channels contribute to action potential-induced calcium current in the soma and dendrites of rat lamina I neurons. Adapted with permission from Harding et al. [27]

However, there is still some debate regarding the exact subpopulations of primary afferent neurons that express T-type channels, and more specifically Ca_v_3.2. Electrophysiological experiments have consistently confirmed the presence of T-type channels in isolated and cultured primary afferent neurons, predominantly within small and medium-size cells, corresponding to C and Aδ-fibers, respectively [59–61]. Other early characterizations also found evidence for T-type channels in some, but not all small and medium-size primary afferent neurons, and that the predominant contributing channel was Ca_v_3.2 [55, 56, 62]. Studies have confirmed that at least some of these neurons corresponded to nociceptors through capsaicin challenge or Isolectin B4 (IB4) positivity, or through confirmation of high threshold mechanoreception [63, 64]. However, other studies have suggested that Ca_v_3.2 is restricted to D-hair mechanoreceptors that correspond to a group of Aδ low threshold mechanoreceptors [65, 66]. While it does appear that Ca_v_3.2 is present in D-hair mechanoreceptors, converging evidence now indicates it is also present in other types of primary afferent neurons such as C fiber nociceptors and low threshold mechanoreceptors, and Aδ fiber nociceptors (Fig. 2) [26, 67, 68].

Supporting this, a wealth of studies now indicate that Ca_v_3.2 is upregulated within rodent primary afferent neurons in many chronic inflammatory and neuropathic pain conditions, and this has been thoroughly reviewed by Cai et al. [69]. These studies highly implicate primary afferent neuron Ca_v_3.2 as being pronociceptive and contributing to hyperexcitability of nociceptive circuitry in chronic pain conditions. Indeed, selective knockdown using intrathecal delivery of oligodeoxynucleotides (ODNs) of each T-type channel subtype within lumbar DRGs and spinal cord revealed that selective depletion of Ca_v_3.2 expression significantly increases mechanical and thermal pain thresholds in both a model of neuropathic pain and in naïve rodents, but not depletion of Ca_v_3.1 or Ca_v_3.3 [56]. This was confirmed in two other studies that found that knockdown of Ca_v_3.2 using ODNs provided significant analgesia in a model of diabetic peripheral neuropathy [70] and a model of irritable bowel syndrome [71]. In a more recent study, selective knockout of Ca_v_3.2 from primary afferent neurons expressing the VGSC Na_v_1.8 (a VGSC selectively expressed in C fibers) resulted in significantly increased mechanical pain thresholds in both a model of neuropathic pain and in naïve rodents [67]. The finding that Ca_v_3.2 within primary afferent neurons contributes to both chronic pain and acute pain sensation is particularly interesting given that antinociceptive effects are not always observed in Ca_v_3.2 global null mice, likely due to compensation [26, 56, 65, 72], but see [26, 73].

Although DRG ODN depletion experiments by Bourinet et al. suggest that the contributions of Ca_v_3.1 and Ca_v_3.3 in DRG neurons to neuropathic pain are limited [56], other studies have challenged this notion [57, 74]. For example, one study found that Ca_v_3.1 global null mice show less allodynia in a neuropathic pain model, although it is possible that this could be due to loss of contributions by Ca_v_3.1 to allodynia in other areas of the ascending pain pathway, including in spinal cord neurons and brain regions including thalamocortical circuitry [28, 74, 75]. Supporting this possibility, an additional study found that spinal nerve injury led to an increase in mRNA of both Ca_v_3.2 and Ca_v_3.3 specifically in the DRG, whereas Ca_v_3.1 could not be detected [57]. It is therefore clear that our understanding of how T-type channels within primary sensory neurons contribute to pain perception is not complete. Future studies using novel genetic tools and techniques will further delineate the specific contributions of T-type channels to acute and chronic pain especially with respect to Ca_v_3.1 and Ca_v_3.3 channels whose roles are less well understood.

It should also be noted that a recent study found T-type channel current in cultured DRGs from human samples to be significantly smaller than that of rodent DRGs and that these currents exhibit kinetics that are uncharacteristically slow [76]. While this certainly does not preclude the possibility that T-type channels become upregulated and contribute to chronic pain in humans, it underscores the need to further study these mechanisms in primate and human tissue wherever possible. To this end, Li et al. validated their findings of Ca_v_3.2 involvement in chemotherapy-induced peripheral neuropathy in human DRGs [58].

In conclusion, T-type channels are expressed broadly in primary afferent neurons in rodents. While it is still unclear exactly how many distinct populations of primary afferent neuron contain T-type currents, converging evidence indicates that T-type channels contribute to primary afferent neuron excitability and hyperexcitability in chronic pain conditions. In rodent models of chronic pain, there is strong evidence that Ca_v_3.2 within these neurons is upregulated, and contributes to the pathophysiology of chronic pain, with potential additional contributions by Ca_v_3.1 and Ca_v_3.3. Although the link between primary afferent T-type channels and chronic pain is well established in rodents, future studies are still needed to bridge the gap to primates and humans.

## Evidence for primary afferent axonal T-type channels

In addition to their presence in the DRG, T-type channels are also present on the axons of primary afferent neurons. Specifically, Ca_v_3.2 has also been localized on primary afferent axons [62, 67, 77], and even on distal nerve endings within the skin (Fig. 2) [62, 67, 78]. Intraplantar injection of T-type antagonists has been found to be analgesic in several studies, supporting that these axonal T-type channels may play a pronociceptive role [61, 79–81].

T-type channels have also been identified within the spinal cord at primary afferent terminals that synapse onto superficial dorsal horn neurons, where their inhibition significantly reduces neurotransmitter release [17, 67, 68]. Use of a selective Ca_v_3.2 primary antibody suggested that the T-type channel present at these presynaptic terminals into the superficial dorsal horn were Ca_v_3.2 [17], and this was confirmed with Transmission Electron Microscopy [67]. Although the exact role of these presynaptic T-type channels is not yet understood, given their location within the superficial dorsal horn it seems likely that they contribute to nociception and that their inhibition may reduce the transmission of nociceptive information to the brain.

## Contributions of T-type channels to trigeminal neuralgia

Some chronic pain conditions such as trigeminal neuralgia are characterized by changes in the functionality of the trigeminal ganglion (TG) rather than the DRG. Although comparatively less is known about T-type channels within the TG, it does appear to express Ca_v_3.1, Ca_v_3.2, and Ca_v_3.3, with activity of Ca_v_3.2 being increased in TG neurons in a facial inflammatory pain model [82]. Moreover, intra-TG injection of the T-type antagonist TTA-P2, systemic administration of Z944, or silencing of Ca_v_3.2 was shown to produce significant analgesia in rodent models of trigeminal neuralgia [82, 83]. Mutations in Ca_v_3.2 have also been associated with the development of trigeminal neuralgia [84], some of which produce potent gain of function [83]. However, there is also evidence that within the TG, Ca_v_3.1 and Ca_v_3.3 may have significant contributions to chronic pain. Ca_v_3.3 was found to be upregulated in the TG in a rodent model of trigeminal neuralgia [85], and Ca_v_3.1 knockout mice exhibited significantly reduced trigeminal neuropathic pain [86]. Together, these first studies implicate TG T-type channels in at least some trigeminal neuralgia conditions, however more studies are required to determine the relative contributions by each sub-type.

## Evidence for expression of T-type channels in spinal dorsal horn neurons and their modulation in chronic pain conditions

The majority of nociceptive primary afferent terminals synapse into the superficial dorsal horn of the spinal cord, which is comprised of lamina I and II, with a smaller number of projections onto wide dynamic range neurons located in deeper laminae [47, 87]. From here, nociceptive information is processed by a complicated network of excitatory and inhibitory interneurons within the spinal dorsal horn, which give context and allow for integration between other laminae involved in processing of other modalities, including touch, vibration, and itch, and are carried up to the brain by projection neurons largely present within lamina I and V [47, 88–91].

An early in situ hybridization study provided the first concrete evidence that T-type channels are present within the spinal dorsal horn [55]. Transcripts for all three subunits were identified within the dorsal horn, however Ca_v_3.1 and Ca_v_3.2 had the highest expression, with Ca_v_3.2 located more within the superficial dorsal horn. This has been supported by immunohistochemical staining for Ca_v_3.2 and analysis of distribution of Ca_v_3.2-GFP in a genetic knock-in mouse [17, 58, 67, 92].

Several electrophysiological studies have identified functional T-type calcium currents within lamina II neurons, which are sensitive to nickel, mibefradil, and TTA-P2 [93–96]. Importantly, these studies agree that T-type channels are present in most, but not all lamina II neurons, suggesting differences in the complement of VGCCs within distinct lamina II subpopulations. Further classification based on protein markers for different subpopulations have revealed that Ca_v_3.2 is present in both excitatory and inhibitory interneurons, including subpopulations known to synapse onto lamina I projection neurons [29, 91]. T-type channel expression in these neurons increases their excitability, as demonstrated by reduction of neuronal firing of lamina II neurons upon pharmacological inhibition of T-type channels or genetic ablation of Ca_v_3.2 [27, 29, 93, 95].

Within lamina I neurons, functional T-type currents have also been identified, as confirmed through Z944 inhibition (Fig. 2) [27]. Similar to lamina II, T-type channels appear to be present in most, but not all lamina I neurons, and use of pharmacological inhibitors has demonstrated that T-type channels contribute to neuronal excitability as well as action potential afterdepolarizations [27, 28, 97]. Calcium imaging of the soma of lamina I neurons has previously suggested that T-type channels contribute to action potential firing-induced calcium influx [98]. Recent two-photon calcium imaging during action potential backpropagation confirms this finding and further suggests that T-type channels are present both on the soma and dendritic arbour of lamina I neurons [27]. Interestingly, this is highly consistent with previous studies finding postsynaptic T-type channels to be necessary for the induction of long-term potentiation at synapses from C fiber primary afferents onto lamina I neurons [28, 99], although it is not yet clear which sub-types these may be. Given that synaptic plasticity is thought to underlie some of the symptoms of chronic pain, this provides a potential mechanism through which inhibition of spinal cord neuron T-type channels could provide pain relief [52, 53, 100–102].

Compared to the extensive studies defining the upregulation and involvement of T-type channels in primary afferent neurons in many chronic pain models, there are very few studies investigating whether T-type channel distribution or functionality is upregulated in chronic pain conditions. Thus far, only three studies have investigated spinal dorsal horn neuron T-type channel expression in chronic pain models, one reporting upregulation of Ca_v_3.2 and Ca_v_3.3, but not Ca_v_3.1 within the superficial dorsal horn in a rodent model of neuropathic pain [103], and another describing upregulation of Ca_v_3.2 within the superficial dorsal horn in a model of knee osteoarthritis [104]. A final study found that neuropathic pain induced by chronic compression of the lumbar DRG increased spinal expression of Ca_v_3.2 and Ca_v_3.3, but not Ca_v_3.1. This study further revealed that intrathecal (i.t.) delivery of ODNs against Ca_v_3.2 and Ca_v_3.3 relieved neuropathic pain symptoms, including allodynia [105]. Thus, preliminary findings indicate that similar to DRG neurons, spinal T-type channel expression is increased in several models of chronic pain. It is not yet clear which channels are the most prominent contributors, but Ca_v_3.2 and Ca_v_3.3 appear to be involved [74, 104–106].

Indeed, upregulation of spinal T-type channels is consistent with a previous in vivo electrophysiology study that found that the T-type antagonist ethosuximide reduced excitability of superficial dorsal horn neurons, and this effect was even greater in neurons from neuropathic pain rodents [107]. Several studies have found that i.t. administration of T-type antagonists such as nickel, ethosuximide, or mibefradil produce analgesia in a number of inflammatory and neuropathic pain models [71, 92, 108]. However, given the experimental paradigm it is difficult to discern if this reduction in excitability can be ascribed to block of presynaptic primary afferent or postsynaptic dorsal horn T-type channels.

In summary, there is now ample evidence that T-type channels are present in neurons within both lamina I and II of the superficial dorsal horn, and that these channels contribute to neuronal excitability. There appears to be some subpopulation specificity in which neurons express T-type channels, and future studies should investigate this possibility. It also remains unclear to what extent T-type channel upregulation within spinal neurons drives chronic pain symptoms, and similar knockdown experiments as to those performed in DRG neurons could provide this valuable information.

## T-type channel contributions to pain perception and modulation in the brain

Although most studies focus on the potential of pain relief via inhibition of T-type channels on primary afferent neurons or in spinal dorsal horn neurons, many structures within the brain are also crucial for the perception and modulation of pain, including the somatosensory cortex, amygdala, thalamus, anterior cingulate cortex (ACC), and periaqueductal gray (PAG) [48, 50, 109, 110]. Emerging evidence suggests that inhibition of T-type channels within some of these areas may also play a role in analgesia, as observed when T-type channel inhibitors are systemically administered. For example, one study has found T-type channels to be highly localized to GABA neurons present in the PAG, where they contribute to low threshold spiking. Specific knockdown of Ca_v_3.1 within the PAG led to loss of low threshold spikes in these neurons and subsequently impaired opioid-induced analgesia [44].

In another study, Ca_v_3.2 was shown to be upregulated in the ACC in a chronic constriction injury model of neuropathic pain, and this was accompanied by a greater calcium current when recording from ACC neurons. Microinjection of the T-type inhibitor NNC 55-0396 into the ACC produced analgesia [111]. Finally, as mentioned in the introduction, T-type channels are abundant in the thalamus, where they contribute to burst firing in the reticular and thalamocortical relaying neurons [35, 36]. Whereas block of these neurons is well known to reduce seizure activity [24, 35], less is known about how inhibition of T-type channels in thalamic neurons may affect pain perception. One study suggests that inhibition of thalamic T-type channels may actually increase pain, at least with respect to visceral pain [112], however others suggest a more traditional role of T-type channels in increasing neuronal excitability, and thus blocking thalamic T-type channels produces analgesia [43, 75].

## T-type channels as therapeutic targets for pain

Understanding the role of T-type channels in pain processing and their modulation in models of chronic pain has long been hindered by the lack of selective T-type antagonists. Early antagonists included amiloride and ethosuximide, with each of these antagonists creating significant off-target effects on other VGCCs or VGSCs [113]. Despite these off-target effects complicating interpretation of results, intraperitoneal (i.p.) administration of ethosuximide produced significant analgesia in multiple rodent neuropathic pain models [114–116]. Similarly, i.p. injection of amiloride also produced significant analgesia in a model of rodent inflammatory pain [117]. It should be noted that within the same study, i.t. administration of amiloride also produced analgesia, giving a first indication that T-type antagonists can produce analgesia at spinal sites of action, although it remains to be discerned whether this action is at presynaptic primary afferent terminals, postsynaptically at spinal dorsal horn neurons, or a combination of both.

In another study, i.p. administration of the nonselective T-type antagonist mibefradil was found to be analgesic [114, 118]. Since mibefradil does not cross the blood–brain barrier, the effects of i.p. administration can be considered to be through peripheral action alone [119]. Another peripherally restricted T-type antagonist, ABT-639, also produced analgesia when administered i.p., albeit only in rodent models of neuropathic but not inflammatory pain [120]. However, in human clinical trials, ABT-639 failed to produce analgesia in patients suffering from diabetic peripheral neuropathy [121, 122], suggesting that in a clinical setting peripherally-restricted T-type antagonists may not be sufficient to produce analgesia. It is possible this is due to human primary afferent neurons not expressing high levels of T-type channels as compared to rodents [76]. Supporting this, i.t. injection of ABT-639 in a model of inflammatory bowel disease did produce significant reductions in pain hypersensitivity, providing further evidence that spinal T-type channels may be an effective pain relief target [123].

Other compounds with known T-type channel inhibition also produce analgesia in vivo, including dihydropyridines and cannabinoids. Although typically considered inhibitors of L-type calcium channels, dihydropyridines (DHPs) including amlodipine can also effectively inhibit T-type channels [124–126], in some cases with high selectivity over L-type channels [127]. In this context, one such DHP based T-type channel inhibitor was shown to attenuate both inflammatory and neuropathic pain in mice [128]. Similarly, the well-known cannabinoids delta-9-tetrahydrocannabinol (Δ9-THC) and cannabidiol (CBD) produce marked state-dependent inhibition of all T-type channels, but especially that of Ca_v_3.1 and Ca_v_3.2 [129, 130]. The endogenous cannabinoid anandamide and some lipoamino acids have also shown efficacy in inhibiting T-type channels in vitro [131–133], along with several synthetic cannabinoid receptor agonists [131, 134]. When tested in vivo, inhibition of Ca_v_3.2 by each of the two lipoamino acids, N-arachidonoyl glycine and N-arachidonoyl 3-OH-γ-aminobutyric acid increased thermal pain threshold in naïve rodents, but no chronic pain models were tested. Although there are many potential targets through which these lipoamino acids could be acting, this increase in thermal pain threshold was not observed in Ca_v_3.2 global null mice, suggesting that the analgesic effect was mediated by Ca_v_3.2 [132]. Indeed, rational design of T-type channel antagonists from cannabinoids have produced novel mixed cannabinoid receptor agonists/T-type channel antagonists with significant analgesic efficacy in both rodent inflammatory and neuropathic pain models [135–138]. Although more work is needed to determine to what extent Ca_v_3.1 channel inhibition by cannabinoids can also produce analgesia, together these results indicate that both cannabinoid receptor agonism and T-type channel antagonism are effective means to treat chronic pain.

**Fig. 3:**
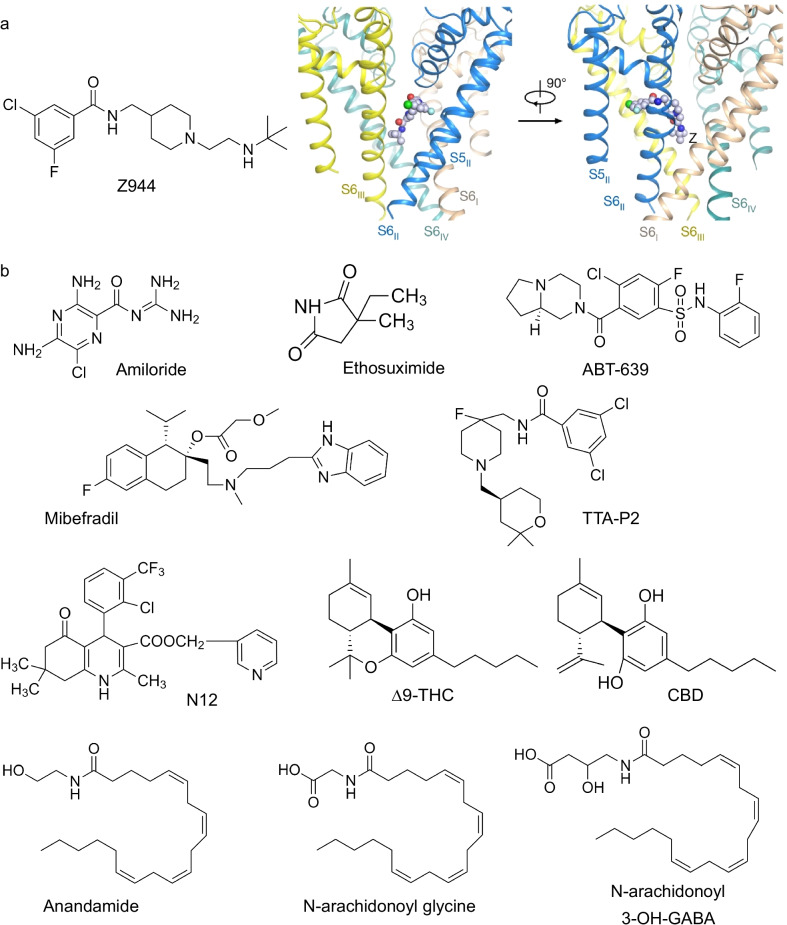
Known inhibitors of T-type channels with analgesic properties. **a** Left: structure of the high affinity and selectivity T-type channel inhibitor Z944. Right: Z944 shown within the binding pocket of Ca_v_3.1. Adapted with permission from Zhao et al. [16]. **b** Structures of commonly utilized T-type channel inhibitors, with varying degrees of affinity and selectivity. Many of these compounds mediate analgesia in preclinical pain models

Newer compounds have since been developed with much greater selectivity for T-type channels and blood–brain barrier permeability, including TTA-P2 and Z944 (Fig. 3) [35, 139]. Use of these next generation antagonists has further indicated that inhibiting T-type channels produces analgesia. For example, i.p. administration of TTA-P2 produced analgesia in both inflammatory and neuropathic pain models [140], and i.p. administration of Z944 produced analgesia in an inflammatory model of pain [27]. Notably, comparison of analgesia produced by Z944 between males and females did not indicate any sex differences, suggesting that global T-type channel inhibition is effective for treatment of pain regardless of sex [27].

Despite the advances made in specificity and selectivity with newer generation T-type channel inhibitors like Z944, potential off-target effects for patients remain a concern. The recent resolution of the cryo-EM structure of Ca_v_3.1 alone and in complex with Z944 represents a significant step forward in our understanding of the structure of Ca_v_3.1 and T-type channels in general (Fig. 3) [16]. Specifically, knowing that Z944 interacts with Ca_v_3.1 to produce inhibition of the channel both through pore block and allosteric modulation may explain how this compound produces such effective inhibition, and its interaction with amino acid residues that are selectively found in Ca_v_3 channels explains the greater selectivity of Z944 for Ca_v_3 channels over HVA channels. These findings may serve as a basis for future development of T-type channel inhibitors with enhanced selectivity, or perhaps even the development of sub-type selective inhibitors.

In summary, while the above studies do not define an exact anatomical locus for how T-type channel inhibition produces analgesia, they clearly and consistently demonstrate that blocking T-type channels produces potent analgesia in rodent models of chronic pain, and that this effect is observed in both males and females.

## Targeting and treating upregulation of T-type channels in chronic pain

The trafficking and function of T-type channels can be modulated by at least three post-translational modifications: N-linked glycosylation, phosphorylation, and ubiquitination. The bulk of studies thus far have been completed with Ca_v_3.2, and thus will be the focus of this section.

Glycosylation involves the addition of sugar groups to extracellularly facing asparagine residues on the Ca_v_3 protein, and is believed to increase the surface expression of Ca_v_3 by promoting proper protein folding and removal from the endoplasmic reticulum [141]. Among four candidate asparagines, two were found to be particularly important for this process—N192 was found to be a potent regulator of channel expression, whereas N1466 regulated channel activity [141]. Indeed, blocking glycosylation of Cav3.2 in models of peripheral diabetic neuropathy was sufficient to reduce increased Ca_v_3.2 currents and pain, without affecting normal Ca_v_3.2 current or pain thresholds in naïve mice [142, 143]. There is also evidence that Ca_v_3.1 channels can undergo glycosylation [144], and would be interesting to study in the context of pain.

Although thus far relatively unexplored, phosphorylation of T-type channels could be another mechanism through which channel activity increases in chronic pain. One study has found that expression of cyclin dependent kinase 5 (CDK5) increases Ca_v_3.2 current in vitro, and that administration of a CDK5 inhibitor decreases T-type current in cultured DRG neurons [145]. Supporting a role in upregulation of Ca_v_3.2 function in chronic pain, CDK5 was found to be increased in a neuropathic pain model, and i.t. administration of a CDK5 inhibitor partially reversed the pain phenotype [145]. Although only tested thus far in HEK-293 cells, there is also evidence that CDK5 can also increase current density of Ca_v_3.1 through phosphorylation [146]. Future studies could further investigate the potential role of phosphorylation in inflammatory pain models, and whether this type of T-type channel phosphorylation occurs in spinal dorsal horn neurons.

Finally, ubiquitination has also been found to regulate T-type channels. Ubiquitination can regulate protein trafficking by increasing the likelihood for a protein to be targeted for degradation through addition of one or more ubiquitin groups to consensus lysine residues [147, 148]. Studies have found that Ca_v_3.2 can be ubiquitinated at a specific lysine residue contained within the domain III-IV linker of the channel, and subsequently deubiquitinated by the deubiquitinase USP5 [72]. Importantly, USP5 expression in DRG neurons and spinal cord is upregulated in both inflammatory and neuropathic models of chronic pain [72, 149]. In additional experiments, electrophysiological recordings of postsynaptic excitatory currents in dorsal horn lamina II neurons confirmed that blocking USP5-mediated deubiquination of Ca_v_3.2 led to an increased paired-pulse ratio, indicative of increased neurotransmitter release probability, and therefore increased presynaptic Ca_v_3.2 expression. Together these experiments indicate that USP5 modulates Ca_v_3.2 expression at presynaptic primary afferent synapses into the spinal dorsal horn. However, based on immunostaining it is likely that USP5 is also upregulated in spinal dorsal horn neurons in chronic pain models where it may dysregulate Ca_v_3.2 channels [72].

Blocking USP5-mediated deubiquitination of Ca_v_3.2 is analgesic in a number of chronic pain models, and across both sexes [72, 150–152]. This can potentially be explored for the purpose of pain therapeutics, since small organic disruptors of the USP5-Ca_v_3.2 interaction are analgesic in rodent models of inflammatory and neuropathic pain [149, 153]. In addition, USP5 regulation of Ca_v_3.2 itself is under control of post translational modification such as by SUMOylation [154], and the upregulation of USP5 appears to be dependent on neuronal activity, such that non invasive optogenetic stimulation of primary afferents leads to an increase in USP5 expression in DRG neurons, along with a transient USP5 /Ca_v_3.2 dependent behavioral sensitization [155].

Together, these three mechanisms serve to alter the trafficking and function of Ca_v_3.2 in DRG neurons in a number of chronic pain conditions. Regulation of any of these processes through small molecule inhibitors holds the potential for producing analgesia with fewer side effects, given that they do not appear to alter normal nociception [149]. If these same mechanisms hold true within spinal dorsal horn neurons (and with Ca_v_3.1 and Ca_v_3.3), it could also provide multiple sites of action upon which T-type channel inhibitors and small molecule disruptors of T-type channel trafficking could provide relief from chronic pain.

## Concluding remarks and perspective

Despite the wealth of evidence that inhibition of T-type channels produces analgesia, remaining questions within the field include the endogenous role of T-type channels in nociception and pain, the precise cellular locus at which T-type inhibitors mediate their analgesic actions and the translatability of preclinical findings to the human clinical population. Nevertheless, the high degree of efficacy of T-type channel antagonists in relieving pain symptoms across a wide spectrum of chronic pain conditions highlights their exceptional potential. As described above, the many possible sites of action including those in the periphery, in somata, axons, and spinal presynaptic synaptic terminals of primary afferent neurons, in spinal cord lamina I and II neurons, and within the brain provide a potential explanation for the robust analgesia observed in preclinical models. The notion that there is less expression of Ca_v_3.2 channels in human primary afferent neurons does not preclude the possibility of these channels serving as potential drug targets in human pain conditions, given that these channels are expressed at multiple loci along the pain pathway. In addition, preclinical evidence suggests that T-type channels are a viable target in both males and females, overcoming a major hurdle through which many clinical-stage target compounds fail to cross. Finally, T-type antagonists have shown good tolerability in human clinical trials for epilepsy, and unlike ABT-639, systemic T-type antagonists like Z944 show promise in clinical studies [156]. Future drug development efforts will be aided by cryo-EM structures, such as that of Ca_v_3.1 in complex with Z944 [16], and homology modeling of other Ca_v_3 subtypes. Together, this may help pave the way towards a new palette of T-type calcium channel therapeutics for the treatment of pain.

## Data Availability

Not applicable.
